# The Anti‐Human P2X7 Monoclonal Antibody (Clone L4) Can Mediate Complement‐Dependent Cytotoxicity of Human Leukocytes

**DOI:** 10.1002/eji.202451196

**Published:** 2025-01-24

**Authors:** Amal Elhage, Debbie Watson, Ronald Sluyter

**Affiliations:** ^1^ Molecular Horizons and School of Chemistry and Molecular Bioscience University of Wollongong Wollongong Australia

**Keywords:** complement‐dependent cytotoxicity, therapeutic antibody, *P2RX7*, P2X7 expression, P2X7, purinergic receptor, purinergic signalling

## Abstract

P2X7 is an extracellular adenosine 5′‐triphosphate (ATP)‐gated cation channel that plays various roles in inflammation and immunity. P2X7 is present on peripheral blood monocytes, dendritic cells (DCs), and innate and adaptive lymphocytes. The anti‐human P2X7 monoclonal antibody (mAb; clone L4), used for immunolabelling P2X7 or blocking P2X7 activity, is a murine IgG2_b_ antibody, but its ability to mediate complement‐dependent cytotoxicity (CDC) is unknown. In this study the functionality of this mAb was confirmed by inhibition of ATP‐induced Ca^2+^ responses in HEK‐293 cells expressing P2X7 (HEK‐P2X7). Spectrophotometric measurements of lactate dehydrogenase release demonstrated that the anti‐P2X7 mAb mediated CDC in HEK‐P2X7 but not HEK‐293 cells. Further, flow cytometric measurements of the viability dye, 7‐aminoactinomycin D, showed that this mAb mediated CDC in human RPMI 8226 but not mouse J774 cells. Immunolabelling with this mAb and flow cytometry revealed that relative amounts of cell surface P2X7 varied between human peripheral blood leukocytes. As such, the anti‐P2X7 mAb preferentially mediated CDC of leukocytes that displayed relatively high cell surface P2X7, namely monocytes, DCs, natural killer T cells, myeloid‐derived suppressor cells, and T helper 17 cells. Together, this data highlights a novel approach to target cellular P2X7 and to limit unwanted P2X7 functions.

## Graphical abstract

The anti‐P2X7 monoclonal antibody (clone L4) mediates complement‐dependent cytotoxicity of human leukocytes. This process preferentially targets leukocytes with high cell surface P2X7, such as monocytes and dendritic cells, presenting a new strategy to reduce P2X7 immune functions.

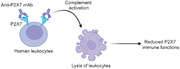

## 1 Introduction

P2X7 is a trimeric ligand‐gated ion channel that is activated by extracellular adenosine 5′‐triphosphate (ATP) to induce the flux of Ca^2+^, Na^+^, K^+^, and organic ions across the cell membrane [1]. P2X7 is involved in a range of physiological processes including inflammation and immunity [2]. This makes the receptor an attractive therapeutic target in many disease settings [3]. P2X7 is present on most cells of the innate and adaptive immune system including dendritic cells (DCs), macrophages, monocytes, and innate and adaptive lymphocytes [4].

A species‐specific human P2X7 monoclonal antibody (mAb; clone L4), generated by Buell and colleagues in 1998, can bind human but not mouse P2X7 nor human P2X1 or P2X4 [5]. Collation of individual flow cytometric studies of human peripheral blood mononuclear cells (PBMCs) using this mAb, suggests a rank order (highest to lowest) of P2X7 on monocytes, DCs, natural killer (NK) cells, B cells, and CD4^+^ and CD8^+^ T cells [6, 7, 8, 9, 10]. However, a comprehensive single analysis of P2X7 expression on human blood mononuclear leukocyte populations with this mAb, or any other anti‐P2X7 mAb, has not been shown to date. In addition to immunolabelling, this mAb can immunoprecipitate human P2X7 from P2X7‐transfected myeloma and HEK‐293 cells [5, 6]. This mAb can also block human P2X7 activity including ATP‐induced channel activity and pore formation [5, 6], ATP‐induced IL‐1β release [5], and P2X7‐mediated phagocytosis [11, 12]. More recently, our group has used this mAb to demonstrate the role of donor (human) P2X7 in promoting graft‐versus‐host disease (GVHD) in humanised mice [13].

The complement pathway is a critical part of the immune system that serves in the defence against foreign and altered host cells [14]. The complement pathway is comprised of a network of over 30 plasma proteolytic enzymes that behave in a cascade‐like manner. This pathway can be activated via three distinct pathways, which converge to form membrane attack complexes, which cause lysis of target cells [15]. This process is commonly referred to as complement‐dependent cytotoxicity (CDC), a term that first originated in 1968 [16]. Of importance to the current study is the classical complement pathway, which is activated by the binding of the complement C1 complex with regions of IgG or IgM antibody–antigen complexes. Whether CDC can be mediated by a P2X7 biologic has not been reported. Given that the anti‐P2X7 mAb (clone L4) is of the IgG2_b_ antibody subclass, which has the potential to mediate CDC [17], it was hypothesised that this mAb could mediate this process in P2X7‐expressing leukocytes.

The current study demonstrates that the anti‐P2X7 mAb (clone L4) can induce CDC in HEK‐293 cells expressing P2X7 (HEK‐P2X7) but not HEK‐293 cells, and in human RPMI 8226 but not mouse J774 cells. Further, immunolabelling with this mAb revealed that relative amounts of cell surface P2X7 varied between human blood leukocytes. As such the anti‐P2X7 mAb favourably mediated CDC of leukocytes that displayed relatively high cell surface P2X7, specifically monocytes, DCs, NK T cells, myeloid‐derived suppressor cells (MDSCs), and T helper (Th17) cells.

## 2 Results

### 2.1 The Anti‐Human P2X7 mAb Inhibits Ca^2+^ Responses in HEK‐P2X7 Cells

**FIGURE 1:**
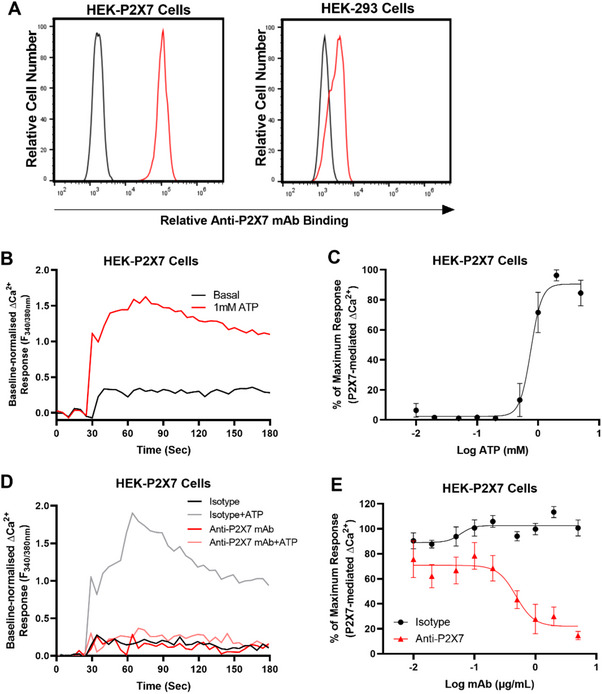
The anti‐human P2X7 mAb inhibits Ca^2+^ responses in HEK‐P2X7 cells. (A) HEK‐P2X7 and HEK‐293 cells were incubated with DyLight488‐conjugated anti‐P2X7 (red line) or isotype control (black line) mAb and analysed by flow cytometry (*n* = 1). (B, C) HEK‐P2X7 cells, pre‐loaded with Fura‐2 AM, were incubated in the presence of increasing concentrations of ATP at 37°C for 3 min (*n* = 3). (D, E) HEK‐P2X7 cells, pre‐loaded with Fura‐2 AM, were pre‐incubated with 0.01 to 5 µg/mL of the anti‐P2X7 (red line) or isotype control mAb (black line) at 37°C for 10 min. Cells were then incubated in the presence of 760 µm ATP for a further 3 min (*n* = 8). (B, D) Ca^2+^ response traces were normalised to baseline (0–30 s) and the area under the curve from 100 to 180 s was used as a measure of the P2X7‐mediated Ca^2+^ flux. (C, E) Responses were normalised to maximum ATP response in each experiment. Data are represented as mean ± SEM. Symbols represent varying concentrations.

To confirm the functionality of the anti‐P2X7 mAb, the ability of this mAb to block ATP‐induced Ca^2+^ responses in HEK‐P2X7 cells was examined. First, to confirm the presence of human P2X7 on HEK‐P2X7 cells, cells were incubated with fluorochrome‐conjugated anti‐P2X7 (clone L4) and isotype control (MPC‐11) mAbs and analysed by flow cytometry with a consistent gating strategy as demonstrated in Figure S1. Non‐transfected HEK‐293 cells, which do not express P2X7 [18], were also examined for comparison. Compared with the isotype control mAb, the anti‐P2X7 mAb showed a right shift in fluorescence for HEK‐P2X7 but not HEK‐293 cells (Figure 1A). The mean fluorescence intensity (MFI) for the anti‐P2X7 mAb on HEK‐P2X7 cells was 98,291. Conversely, the MFI for anti‐P2X7 mAb on HEK‐293 cells was like that of the isotype control mAb values, 3,063 and 1,625, respectively.

Prior to mAb blockade studies, the half maximal effective concentration (EC_50_) for the P2X7‐mediated Ca^2+^ response in HEK‐P2X7 cells was determined. Cells were incubated with increasing concentrations of ATP and Ca^2+^ responses measured using a fluorescent plate reader (Figure 1B,C). ATP induced Ca^2+^ responses in Fura‐2 AM loaded HEK‐P2X7 cells in a concentration‐dependent manner with a maximal response occurring at 2 to 5 mM and an EC_50_ of 759 ± 15 µM (Figure 1C). Finally, to determine if the anti‐P2X7 mAb could inhibit the P2X7‐mediated Ca^2+^ response, Fura‐2 AM loaded HEK‐P2X7 cells were preincubated with increasing concentrations of the anti‐P2X7 or isotype control mAb, and then incubated with 760 µM ATP, approximate to the EC_50_ obtained above (Figure 1D,E). The anti‐P2X7 mAb inhibited ATP‐induced Ca^2+^ responses in HEK‐P2X7 cells in a concentration‐dependent manner with maximum blockade (∼85% inhibition) at 5 µg/mL and with a half maximum inhibitory concentration (IC_50_) of 0.48 ± 0.24 µg/mL (Figure 1E). Conversely, the isotype control showed minimal to no blockade of ATP‐induced Ca^2+^ responses in HEK‐P2X7 cells (Figure 1E).

### 2.2 The Anti‐Human P2X7 mAb Induces CDC in Human P2X7 Expressing Cell Lines

To determine if the anti‐human P2X7 mAb (clone L4) could mediate CDC, HEK‐P2X7 cells were incubated with either Dulbecco's phosphate‐buffered saline (PBS) (mAb diluent), the anti‐P2X7 or isotype control mAb in the presence of complement. Cell cytotoxicity was assessed using a spectrophotometric lactate dehydrogenase (LDH) release assay. Non‐transfected HEK‐293 cells were studied as a negative control. Percent cytotoxicity at 2 h was calculated as a percentage of maximum LDH release (obtained using the provided cell lysis solution).

**FIGURE 2:**
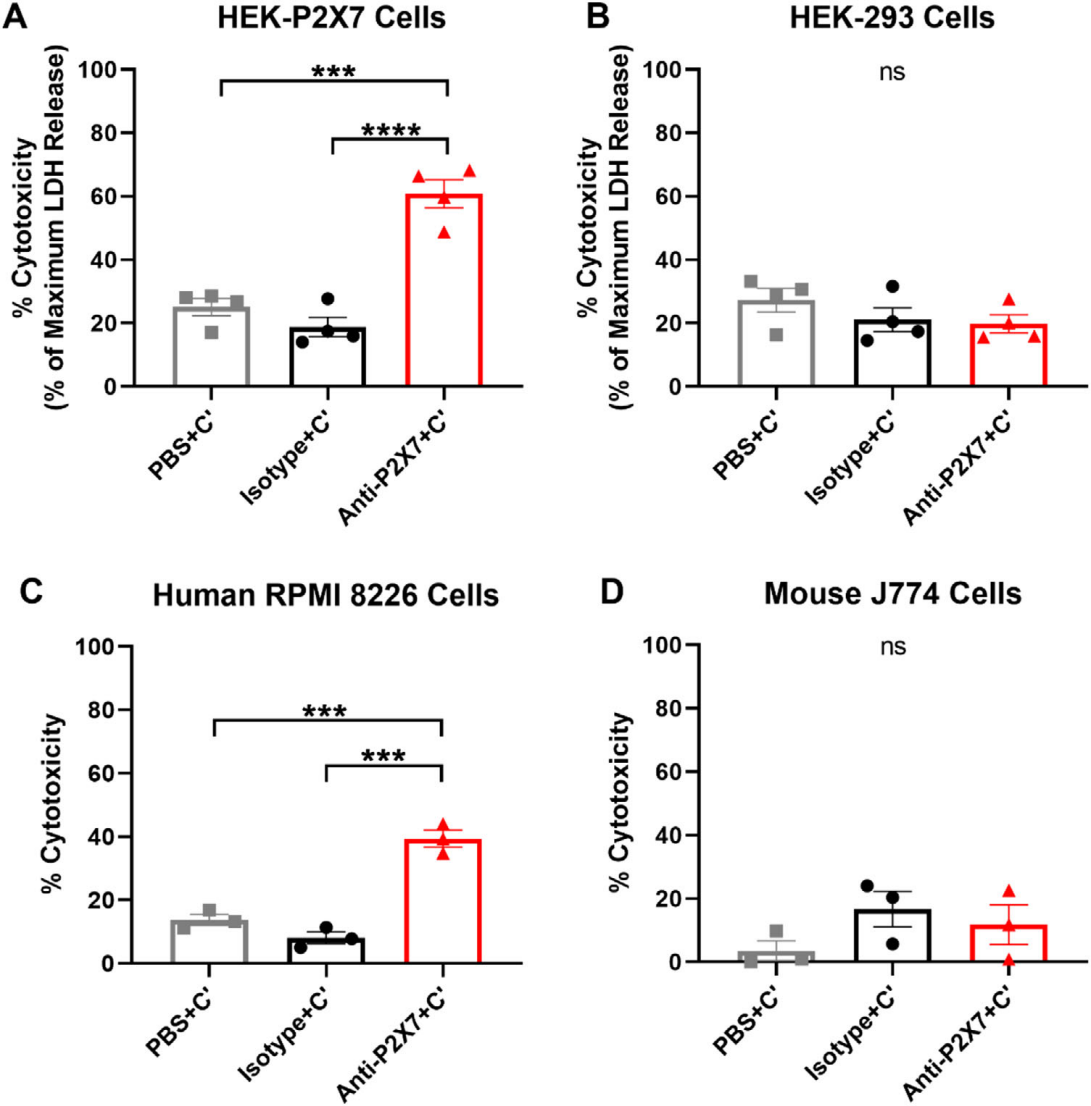
The anti‐human P2X7 mAb induces CDC in human P2X7 expressing cell lines. (A) HEK‐P2X7, (B) HEK‐293, (C) human RPMI 8226 and (D) mouse J774 cells were incubated with PBS, anti‐P2X7 (2 µg/mL) or isotype control mAb (2 µg/mL) in the absence (not shown) or presence of complement (C’) at a final dilution of (A, B) 1:4 or (C, D) 1:8 for 2 h at 37°C. (A, B) Percent cytotoxicity was assessed using a spectrophotometric LDH release assay. Percent cytotoxicity was calculated as a percentage of maximum LDH release (*n* = 4). (C, D) Percent cytotoxicity was determined using 7AAD and flow cytometry, with cells incubated in the absence of complement subtracted from cells incubated with complement (*n* = 3). (A–D) Data represented as mean ± SEM. Significance was assessed by a one‐way ANOVA. ^***^ *p *< 0.001; ^****^ *p *< 0.0001; no significant difference (ns). Symbols represent individual experiments.

HEK‐P2X7 cells incubated in the presence of PBS or isotype control mAb showed similar mean cytotoxicities of 25.1 ± 2.7% and 18.8 ± 3.1%, respectively (Figure 2A). HEK‐P2X7 cells incubated in the presence of the anti‐P2X7 mAb revealed a mean cytotoxicity of 60.8 ± 4.4%, which was significantly greater than that of cells incubated in the presence of PBS (*p *< 0.001) and the isotype control mAb (*p *< 0.0001; Figure 2A). In contrast, HEK‐293 cells incubated in the presence of PBS, isotype control or the anti‐P2X7 mAb revealed similar cytotoxicities of 27.2 ± 3.8%, 21.0 ± 3.7%, and 19.8 ± 2.8%, respectively, with no significant differences observed between treatments (Figure 2B).

Next, the ability of the anti‐P2X7 mAb to mediate CDC was assessed using a flow cytometric cell death assay with human RPMI 8226 cells, known to express endogenous cell surface P2X7 [19]. Mouse J774 cells, which also express cell surface P2X7 [20], were studied as a negative control. Cells were incubated as above, but with cell viability at 2 h assessed using a viability dye, 7‐aminoactinomycin D (7AAD), and flow cytometry. The gating strategy used to identify live and dead cells is demonstrated in Figure S2. Human RPMI 8226 cells incubated in the presence of PBS or isotype control mAb showed mean cytotoxicities of 13.7 ± 1.7% and 8.1 ± 1.9%, respectively (Figure 2C). RPMI 8226 cells incubated in the presence of the anti‐P2X7 mAb revealed a mean cytotoxicity of 39.4 ± 2.7%, which was significantly greater than that of cells incubated in the presence of PBS (*p* < 0.001) or the isotype control mAb (*p *< 0.001) (Figure 2C). In contrast, mouse J774 cells incubated in the presence of PBS, isotype control or the anti‐P2X7 mAb revealed low amounts of cytotoxicity of 3.6 ± 3.1%, 16.7 ± 5.6%, and 11.8 ± 6.2%, respectively, with no significant differences observed between treatments (Figure 2D).

### 2.3 Cell Surface P2X7 is Present on Human Blood Mononuclear Leukocyte Subsets

**FIGURE 3:**
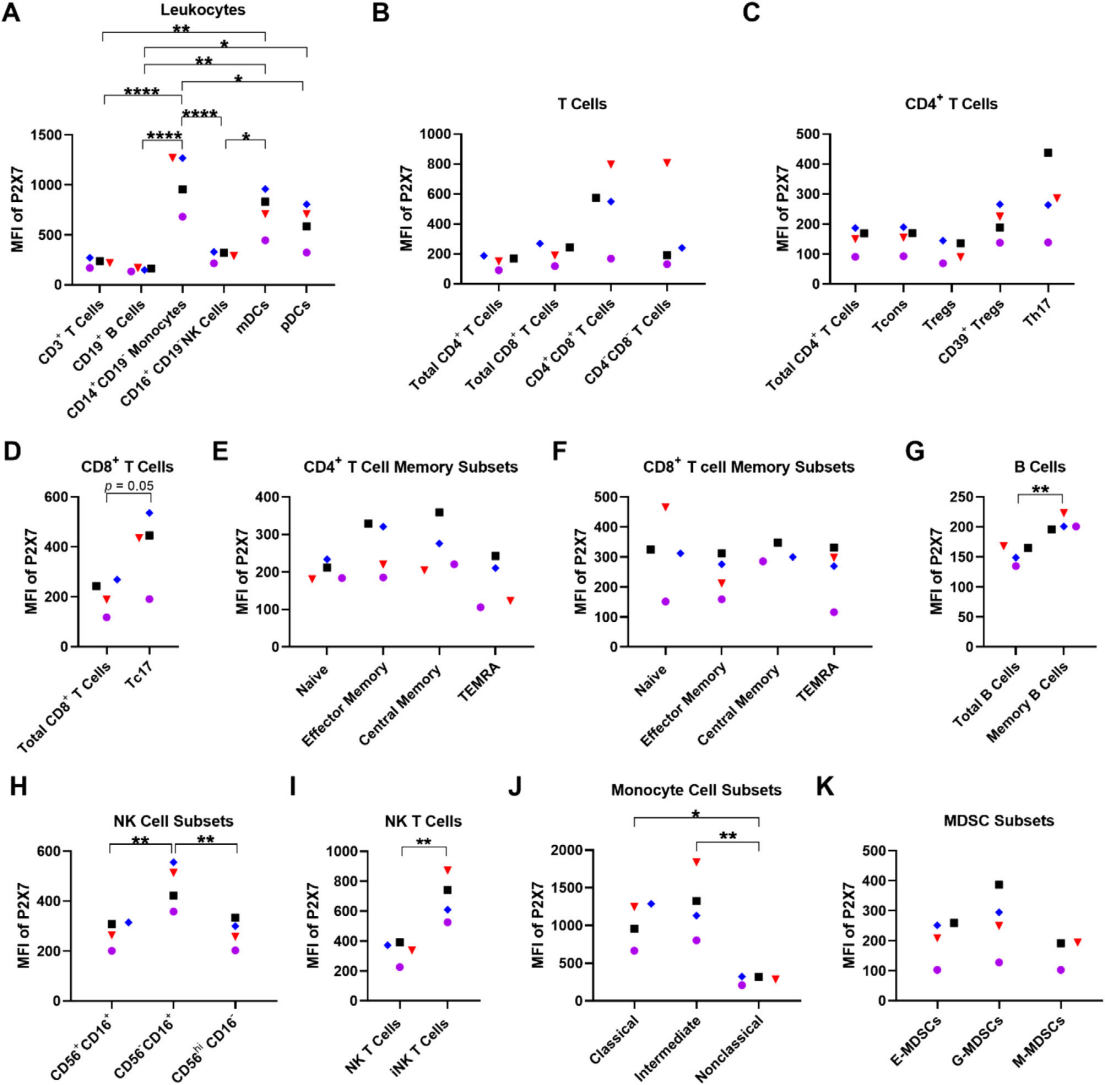
Cell surface P2X7 is present on human blood mononuclear leukocyte subsets. (A–K) Human PBMCs, isolated by Ficoll‐Paque density gradient centrifugation, were labelled with fluorochrome‐conjugated mAbs. Flow cytometric analyses were performed. Sequential flow cytometric gates were selected using a consistent gating strategy as demonstrated in Figures S3–S6. Cell subsets were examined for relative P2X7 expression. Relative expression was determined as the difference in MFI of anti‐P2X7 mAb MFI minus the respective FMO control (*n* = 4 independent donors). Symbols represent individual donors. Statistical analyses were conducted using a (A, C, E, F, H, J) one‐way ANOVA, (B, K) Kruskal–Wallis or (D, G, I) unpaired *t‐*test. ^*^ *p *< 0.05; ^**^ *p *< 0.01; ^****^ *p *< 0.0001.

Previous studies with the anti‐human P2X7 mAb (clone L4) or the anti‐human P2X7 nanobody (Dano1) have shown that P2X7 is present on various human mononuclear blood leukocyte subsets [6, 7, 8, 9, 10, 21]. Before determining whether the anti‐P2X7 mAb can mediate CDC in these cells, the presence of cell surface P2X7 on blood mononuclear leukocyte subsets was first examined. Human PBMCs from four healthy donors were labelled with fluorochrome‐conjugated mAbs (Table S1) and analysed by flow cytometry using consistent gating strategies as demonstrated in Figures S3–S6. Relative cell surface P2X7 expression was determined as the difference between the MFI of anti‐P2X7 mAb and the MFI of the corresponding fluorescence minus one (FMO) control. P2X7 was present on all blood mononuclear leukocyte subsets analysed (Figure 3).

The relative cell surface P2X7 expression (reported as mean MFI ± SEM) was first compared on the main subtypes of PBMCs. This analysis revealed a rank order of expression (highest to lowest) of CD14^+^ monocytes (1,044.0 ± 141.3), myeloid DCs (mDCs) (736.3 ± 109.7), plasmacytoid DCs (pDCs) (606.0 ± 104.7), CD16^+^ NK cells (288.5 ± 25.6), CD3^+^ T cells (223.8 ± 21.3), and CD19^+^ B cells (154.3 ± 7.7; Figure 3A). CD14^+^ monocytes had significantly greater cell surface P2X7 expression compared with CD3^+^ T cells (*p *< 0.0001), CD19^+^ B cells (*p *< 0.0001), CD16^+^ NK cells (*p *< 0.0001), and pDCs (*p* = 0.02). mDCs had significantly greater cell surface P2X7 expression compared with CD3^+^ T cells (*p* = 0.006), CD19^+^ B cells (*p* = 0.002), and CD16^+^ NK cells (*p* = 0.02). Lastly, pDCs had significantly greater cell surface P2X7 expression compared with CD19^+^ B cells (*p* = 0.02).

Next, relative cell surface P2X7 expression was examined on subsets of lymphocytes and monocytes. Amongst CD3^+^ T cells, P2X7 expression was similar on CD4^+^ (149.3 ± 20.8), CD8^+^ (204.8 ± 33.4), and CD4^−^CD8^−^ cell subsets (342.0 ± 156.3), with greater expression on CD4^+^CD8^+^ cells (521.8 ± 130.3), but this did not reach statistical significance (Figure 3B). Analysis of total CD4^+^ T cell subsets revealed similar P2X7 expression between conventional T cells (Tcons) (152.0 ± 20.9), T regulatory cells (Tregs) (110.0 ± 18.2), and CD39^+^ Treg cells (204.5 ± 27.2), with slightly but not significantly greater P2X7 expression on Th17 cells (281.8 ± 61.3; Figure 3C). Analysis of CD8^+^ T cell subsets showed greater expression on T cytotoxic 17 (Tc17) cells (402.0 ± 73.9) compared with total CD8^+^ T cells (204.8 ± 33.4; *p* = 0.05; Figure 3D).

A comparison of naive and memory CD4^+^ and CD8^+^ T cells was also undertaken. CD4^+^ T cell memory subsets showed similar P2X7 expression between naive (202.8 ± 12.5), effector memory (264.0 ± 35.9), central memory (265.3 ± 34.8), and terminally differentiated effector memory (TEMRA) cells (170.8 ± 33.3; Figure 3E). Likewise, CD8^+^ T cell memory subsets showed similar P2X7 expression between naive (313.8 ± 64.2), effector memory (239.8 ± 33.9), central memory (311.3 ± 18.8), and TEMRA cells (254.0 ± 47.7; Figure 3F).

P2X7 expression on memory B cells (205.3 ± 6.0) was significantly greater compared with total B cells (154.3 ± 7.7) (*p* = 0.002; Figure 3G). P2X7 expression was significantly greater on CD56^−^CD16^+^ NK cells (462.3 ± 45.6) compared with both CD56^+^CD16^+^ (272.0 ± 26.2; *p* = 0.008) and CD56^hi^CD16^−^ NK cells (273.8 ± 28.2; *p* = 0.009), which had similar expression (Figure 3H). Notably, P2X7 expression on invariant (i) NK T cells (687.5 ± 75.9), identified as Vα24‐Jα18 T cell receptor^+^, was significantly greater than total NK T cells (331.5 ± 36.9) (*p* = 0.006; Figure 3I). Monocyte cell subsets revealed a rank order of P2X7 expression on intermediate (1275.0 ± 216.6; *p* = 0.003 to non‐classical), classical (1040.0 ± 144.0; *p* = 0.02 to non‐classical) and non‐classical monocytes (284.0 ± 26.4; Figure 3J).

A limited number of studies indicate the presence of P2X7 on mouse MDSCs [22, 23, 24], but whether this receptor is present on human MDSCs has not been reported. Analysis of MDSC subsets revealed the presence of cell surface P2X7 on early (E‐) (206.0 ± 36.1), granulocytic (G‐) (265.0 ± 53.8), and monocytic (M‐) MDSCs (163.0 ± 30.0) with similar expression between all three subsets (Figure 3K).

### 2.4 The Anti‐Human P2X7 mAb Mediates CDC of Human Blood Leukocyte Subsets That Display Relatively High Cell Surface P2X7

To determine if the anti‐P2X7 mAb can mediate CDC in leukocytes, human PBMCs were incubated with either the anti‐P2X7 or isotype control mAb in the presence of complement for 2 h. Cell subsets were analysed by flow cytometry using the same gating strategies as above, with the number of cells quantified using count beads.

**FIGURE 4:**
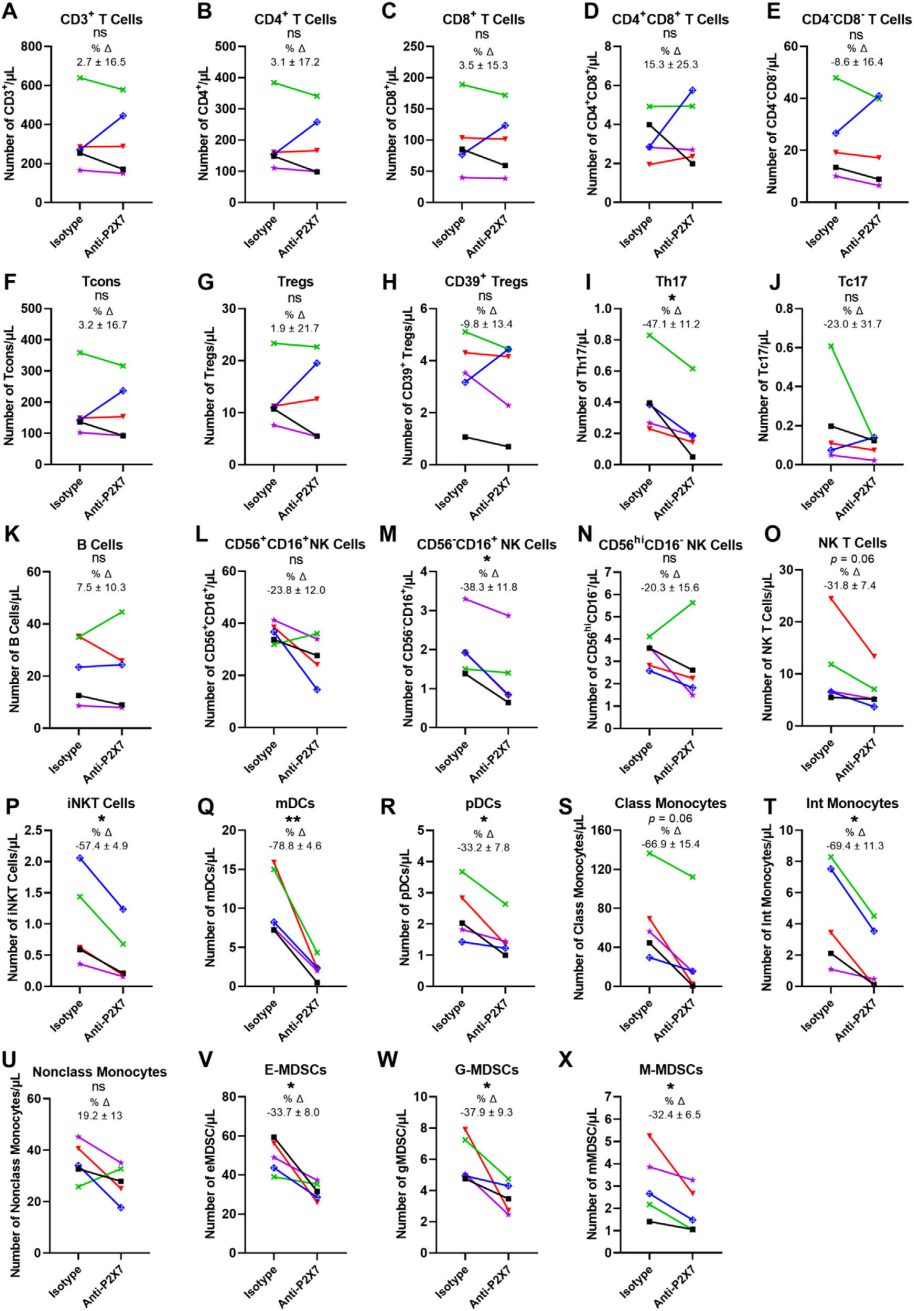
The anti‐human P2X7 mAb mediates CDC of human blood mononuclear leukocyte subsets with relatively high cell surface P2X7. (A–X) Human PBMCs were incubated with anti‐P2X7 (2 µg/mL) or isotype control mAb (2 µg/mL) in the presence of complement (1:8 final dilution) for 2 h at 37°C. Cells were labelled with fluorochrome‐conjugated mAbs. Flow cytometric analyses were performed. Sequential flow cytometric gates were selected using a consistent gating strategy as demonstrated in Figures S3, S5, and S6. Numbers of leukocyte subsets were determined using precision count beads (*n* = 5 independent donors). Data represented as mean ± SEM. Symbols represent individual donors. Significance was assessed by using the paired Student's *t‐*test. ^*^ *p *< 0.05; ^**^ *p *<  0.01; no significant difference (ns). Percent change (% Δ) was assessed by comparing cell numbers in anti‐P2X7 and isotype control mAb wells.

Analysis of T cell subsets showed that numbers of CD3^+^ (*p* = 0.94; Figure 4A), CD4^+^ (*p* = 0.81; Figure 4B), and CD8^+^ T cells (*p* = 0.99; Figure 4C), CD4^+^CD8^+^ (*p* = 0.78; Figure 4D), and CD4^−^CD8^−^ T cells (*p* = 0.85; Figure 4E)_,_ Tcons (*p* = 0.81) (Figure 4F), Tregs (*p* ≥ 0.99; Figure 4G) and CD39^+^ Tregs (*p* = 0.61; Figure 4H) were comparable between treatments with minimal percent change. In contrast, Th17 cell numbers were significantly decreased by 47.1 ± 11.2% (mean ± SEM) in anti‐P2X7 mAb‐treated cells compared with isotype control mAb‐treated cells (*p* = 0.02; Figure 4I). Tc17 cells were partially but not significantly decreased by 23.0 ± 31.7% in anti‐P2X7 mAb‐treated cells compared with isotype control mAb‐treated cells (*p* = 0.31; Figure 4J).

The numbers of B cells were similar between both treatment groups (*p* = 0.85; Figure 4K). CD56^−^CD16^+^ NK cell numbers were significantly decreased by 38.3 ± 11.8% in anti‐P2X7 mAb‐treated cells compared with isotype control mAb‐treated cells (*p* = 0.02; Figure 4M). Conversely, the numbers of both CD56^+^CD16^+^ NK cells (*p* = 0.10; Figure 4L) and CD56^hi^CD16^−^ NK cells (*p* = 0.38; Figure 4N) did not differ between treatment groups. NK T cell numbers were decreased by 31.8 ± 7.4% in anti‐P2X7 mAb‐treated cells compared with isotype control mAb‐treated cells, a difference which approached statistical significance (*p* = 0.06); Figure 4O), while iNKT cells were significantly reduced by 57.4 ± 4.9% in anti‐P2X7 mAb‐treated cells compared with isotype control mAb‐treated cells (*p* = 0.01; Figure 4P).

Both DC subsets also showed a significant reduction in numbers in anti‐P2X7 mAb‐treated cells compared with isotype control mAb‐treated cells, with mDCs decreased by 78.8 ± 4.6% (*p* = 0.006; Figure 4Q) and pDCs decreased by 33.2 ± 7.8% (*p* = 0.02; Figure 4R). Numbers of classical monocytes were decreased by 66.9 ± 15.4% in anti‐P2X7 mAb‐treated cells compared with isotype control mAb‐treated cells, a difference which approached statistical significance (*p* = 0.06; Figure 4S). Intermediate monocyte cell numbers were significantly decreased by 69.4 ± 11.3% between treatment groups (*p* = 0.01; Figure 4T). Conversely, numbers of non‐classical monocytes did not differ between both treatment groups (*p* = 0.14; Figure 4U).

Finally, numbers of all three MDSC subsets were significantly decreased in anti‐P2X7 mAb‐treated cells compared with isotype control mAb‐treated cells, with E‐MDSCs decreased by 33.7 ± 8.0% (*p* = 0.02; Figure 4V), G‐MDSCs decreased by 37.9 ± 9.3% (*p* = 0.03; Figure 4W), and M‐MDSCs decreased by 32.4 ± 6.5% (*p* = 0.04; Figure 4X).

## 3 Discussion

The current study demonstrated that the anti‐human P2X7 mAb (clone L4) can mediate CDC in two human P2X7 expressing cell lines, HEK‐P2X7 and RPMI 8226 cells. Furthermore, it confirmed the cell surface expression of P2X7 on various human blood mononuclear leukocytes and demonstrated for the first time cell surface P2X7 on human MDSCs. Most notably, it demonstrated that this mAb can mediate CDC in human leukocytes expressing relatively high cell surface P2X7.

The current study provides the first evidence that an anti‐P2X7 mAb can mediate CDC. The anti‐P2X7 mAb mediated CDC in HEK‐P2X7 and human RPMI 8226 cells but not (non‐transfected) HEK‐293 cells or mouse J774 cells, confirming that this effect required the presence of human P2X7. Furthermore, this mAb mediated CDC in human monocytes, DCs and iNKT cells, which were observed to express relatively high amounts of cell surface P2X7. Likewise, this effect was seen in MDSCs and Th17 cells (although not Tc17 cells), which expressed relatively high to moderate amounts of cell surface P2X7. In contrast, anti‐P2X7 mAb‐mediated CDC was not observed in the majority of lymphocyte populations, which expressed relatively low amounts of cell surface P2X7. These data indicate that leukocytes that express relatively high amounts of cell surface P2X7 have a greater propensity to undergo anti‐P2X7 mAb‐mediated CDC. Consistent with this notion, anti‐P2X7 mAb‐mediated CDC was observed in classical and intermediate monocytes but not in non‐classical monocytes, which reflected the relative amount of cell surface P2X7 on these subsets. The difference in anti‐P2X7 mAb‐mediated CDC between Th17 and Tc17, which express similar amounts of P2X7, may be due to the cell surface presence of complement component 1 proteins at greater amounts on Th17 cells than other T cell subsets [25].

The capacity of the anti‐P2X7 mAb to induce CDC in human leukocytes may have the potential to selectively deplete P2X7 high‐expressing cells in vitro or in vivo. Thus, this mAb could be used to target these cells in experimental or therapeutic settings to reduce unwanted P2X7 functions. The anti‐P2X7 mAb (clone L4) can reduce GVHD in a humanised NOD‐*scid* IL2Rγ^null^ (NSG) mouse model [13]. This effect was attributed to the blockade of human P2X7 and increased survival of human Tregs. Although this previous study did not rule out a role for this mAb in mediating CDC of human cells, this is unlikely as NSG mice lack complement activity due to a 2‐base pair deletion in the *Hc* gene [26]. Nevertheless, the current study suggests that the anti‐P2X7 mAb could be used to deplete P2X7 high cells, such as donor DCs, ex vivo prior to the transplantation of human leukocytes into NSG mice or humans (for which donor blood stem cell transplantation is indicated), with the goal of reducing GVHD severity. Of note, the use of antibodies and complement for ex vivo cell depletion to mitigate GVHD risk in donor stem cell transplantation has been previously used to reduce GVHD severity [27, 28, 29, 30].

Related to the potential of the anti‐P2X7 mAb mediating CDC in vivo, a phase 1 clinical trial using topical treatment of a polyclonal antibody to non‐functional P2X7 (BIL010t) can reduce the size of primary basal cell carcinoma lesions in 65% of patients [31]. Although the mechanism of action of this antibody was not reported, the findings in the current study suggest that the mechanism of action could be due to CDC. Regardless, P2X7‐mediated CDC could be potentially used in this or other cancers to eliminate P2X7‐expressing tumour cells.

The current study shows the relative cell surface expression of P2X7 on blood mononuclear leukocytes follows a rank order of expression (highest to lowest) on monocytes, DCs, NK T cells, MDSCs, NK cells, CD4^+^ and CD8^+^ T cells, and B cells. Except for MDSCs, for which P2X7 expression has not been reported in humans, this rank order largely aligns with previous studies when combined [6, 7, 8, 9, 10, 21]. Thus, the current study provides the most complete, single analysis of P2X7 expression on blood mononuclear leukocytes to date. In relation to the myeloid cell types, the relative expression on classical, intermediate, and non‐classical monocytes parallels previous findings in control populations of Alzheimer's disease [32, 33]. However, other studies have shown a rank order of P2X7 expression on intermediate, non‐classical, and classical monocytes [10] or no expression of P2X7 on classical and non‐classical monocytes [9]. Regarding DC subsets, a previous study utilising the anti‐P2X7 mAb (clone L4) revealed similar amounts of expression of P2X7 on human blood mDCs and pDCs [9]. However, other studies utilising this mAb showed greater P2X7 expression on mDCs, with little to no expression on pDCs [8, 10]. Reasons for differences in P2X7 expression between some studies and the current study may reflect donor differences. Finally, the current study showed high cell surface expression of P2X7 on iNKT cells, which has been reported in mice, specifically intestinal iNKT cells [34].

During this study, Winzer and colleagues, using an anti‐human P2X7 specific nanobody (Dano1), reported the relative cell surface expression of P2X7 on human blood lymphocytes and total (CD14^+^CD16^+^) monocytes. The former results largely parallel the current findings, with the following exceptions, which again may be due to donor differences. Winzer and colleagues observed significantly greater P2X7 expression of CD4^−^CD8^−^ T cells compared with Tcons, Tregs, and CD8^+^ T cells, which was not observed here. Furthermore, significantly greater P2X7 expression on CD4^+^ effector memory cells compared with CD4^+^ naive and central memory cells was shown previously, which was not observed here.

Finally, the current study showed that the anti‐human P2X7 mAb can block ATP‐induced Ca^2+^ responses in HEK‐P2X7 cells. Inhibition of P2X7‐mediated Ca^2+^ responses by this mAb has not been previously reported; however, this mAb has been shown to block ATP‐induced Ba^2+^ responses (a surrogate of Ca^2+^ responses) in leukemic B lymphocytes [6] as well as ATP‐induced cation dye uptake into various cell types [5, 6, 13]. In the current study, this mAb displayed a concentration‐dependent inhibition of ATP‐induced Ca^2+^ responses, achieving a maximum blockade of approximately 85% at 5 µg/mL and an IC_50_ value of 0.48 ± 0.24 µg/mL. In comparison, previous studies utilising this anti‐P2X7 mAb have reported lower IC_50_ values. The original study of Buell et al. [5] demonstrated an IC_50_ of 0.0008 µg/mL for 2′,3′‐(4‐benzoyl)‐benzoyl‐ATP‐induced nucleotide currents into HEK‐P2X7 cells. While our previous study [13] revealed an IC_50_ of 0.21 µg/mL for ATP‐induced cation dye uptake into human RPMI 8226 cells. These disparities most likely reflect technical differences between the P2X7 activity assays used.

In conclusion, the current study showed that the anti‐human P2X7 mAb (clone L4) can mediate CDC in two human cell lines expressing P2X7. Moreover, this study confirmed the expression of cell surface P2X7 on various human blood mononuclear leukocytes and revealed the presence of P2X7 on human blood MDSCs. Finally, it demonstrated that this mAb can mediate CDC in human leukocytes expressing relatively high cell surface P2X7. Thus, potentially being used in a therapeutic setting by selectively depleting high P2X7 expressing cells in vitro or in vivo to reduce unwanted P2X7 functions.

### 3.1 Data Limitations and Perspectives

This study has several limitations. First, P2X7 mAb‐mediated CDC in human leukocytes was assessed indirectly by measuring changes in the numbers of cell subsets, rather than by measuring cell death directly within these populations. Second, although the anti‐P2X7 mAb was used in the CDC assays at a concentration (2 µg/mL) that induced maximal inhibition of ATP‐induced Ca^2+^ responses in HEK‐P2X7 cells it was not titrated for the CDC assays, which may have resulted in suboptimal mAb‐mediated CDC of some cell types. Finally, studies of P2X7 expression and CDC were conducted using different cohorts of PBMC donors, preventing direct comparisons between these parameters.

This study provides the most comprehensive single analysis of P2X7 expression on human blood leukocyte subsets to date. Further, this study demonstrates for the first time that a biologic targeting P2X7 mediates CDC in cells expressing P2X7 from any species. Use of anti‐P2X7 mAb‐mediated CDC and subsequent depletion of P2X7^+^ cells may represent a new approach to preventing P2X7‐mediated processes in experimental and clinical settings.

## 4 Materials and Methods

### 4.1 Cell Lines

All cell culture reagents were from Thermo Fisher Scientific (Waltham, USA) unless stated otherwise. HEK‐293 cells were from the American Type Culture Collection (Manassas, USA) and were maintained in DMEM/F‐12 medium containing 10% foetal calf serum (FCS), 2 mM GlutaMAX, 100 U/mL penicillin, and 100 µg/mL streptomycin at 37°C and 95% air/5% CO_2_. HEK‐P2X7 cells were originally provided by Dr. Leanne Stokes (University of East Anglia, Norwich, UK) and were maintained in the same medium as HEK‐293 cells but containing 400 µg/mL geneticin. Human multiple myeloma RPMI 8226 cells were from the European Collection of Cell Cultures (Wiltshire, UK). Mouse macrophage J774 cells were obtained from the American Type Culture Collection (Rockville, USA). Both cell lines were maintained in RPMI‐1640 medium containing 10% FCS and 2 mM GlutaMAX. The anti‐P2X7 mAb‐producing mouse hybridoma cell line (clone L4) was originally obtained from the Glaxo Institute for Applied Pharmacology (Cambridge, UK) and maintained in IMDM (Sigma Aldrich, St Louis, MO, USA) containing 20% FCS and 2 mM GlutaMAX. A mouse multiple myeloma cell line producing an IgG2_b_ isotype control mAb (clone MPC‐11) was obtained from CellBank Australia (Westmead, Australia) and maintained in DMEM (Sigma Aldrich) containing 20% horse serum (Sigma Aldrich) and 2 mM GlutaMAX. Cell lines were assessed for *Mycoplasma* spp. contamination using the Myco Alert Mycoplasma Detection Kit (Lonza, Switzerland) as per the manufacturer's instructions and were found to be routinely negative.

### 4.2 Purification of the Anti‐P2X7 and Isotype Control mAbs

Anti‐P2X7 and isotype control mAbs were purified as described [35]. Briefly, tissue culture supernatant (TCSN) was collected from clone L4 and MPC‐11 cell lines. IgG from TCSNs was then purified using a Pierce Protein A Agarose IgG Purification Kit (Thermo Fisher Scientific) according to the manufacturer's instructions. TCSN was incubated with 1 mL Protein A resin for 90 min with gentle rocking. The mixture was re‐packed into a supplied chromatography column and the sample was allowed to flow through to form a bed. The column was washed with the supplied binding buffer, and 1 mL fractions were collected. The absorbances of the fractions were measured at 280 nm using a Suprasil quartz cuvette (Hellma, Müllheim, Germany) in a SpectraMax Microplate Reader spectrophotometer (Molecular Devices, San Jose, CA, USA) to confirm unbound protein was removed from the column. Bound IgG was then eluted with the supplied elution buffer, with 1 mL fractions collected into microfuge tubes containing 50 µL of 1 M Tris solution (pH 9.5) and the absorbances measured as above. Fractions with the highest absorbances, indicating the elution of IgG, were pooled and concentrated to 1 mg/mL in PBS, using a 50 kD molecular cut‐off centrifugal device (Thermo Fisher Scientific) as per the manufacturer's instructions. The purity of IgG preparations was confirmed by protein electrophoresis using 4–10% Mini‐PROTEAN TGX Stain‐Free Protein gels (Bio‐Rad Hercules, CA, USA), which were visualised using a Gel DocTM XR+ Imager (Bio‐Rad).

### 4.3 Human PBMC Isolation

Collection and utilisation of human blood were done in accordance with approval by the University of Wollongong Human Ethics Committee (HE 12/290). PBMCs were isolated by density centrifugation as described [36]. Briefly, peripheral blood was collected from healthy donors (four males and three females; age range 24–52 years) and diluted in an equivalent volume of sterile PBS (Thermo Fisher Scientific). Samples were underlaid with Ficoll‐Paque PLUS (GE Healthcare; Uppsala, Sweden) and centrifuged (560 × *g* for 30 min with deceleration set to 0). PBMCs were recovered from the gradient interface, washed twice with PBS, and resuspended at a final concentration of 1 × 10^6^ cells/mL for P2X7 expression assays or 3 × 10^6^ cells/mL for CDC assays.

### 4.4 Detection of P2X7 on Cell Lines by Immunolabelling and Flow Cytometry

The presence of P2X7 on HEK‐P2X7 and HEK‐293 cells was assessed as described [13]. Briefly, HEK‐P2X7 and HEK‐293 cells were washed in PBS containing 10% FCS (300 × *g* for 5 min). Cells (1 × 10^6^ cells/100 µL) were incubated with DyLight488‐conjugated anti‐P2X7 (clone L4) or isotype control (clone MPC‐11) mAbs, prepared as described [13, 35], and the cell viability dye 7AAD (1 µg/mL; Cayman Chemical, Ann Arbor, USA) for 20 min in the dark on ice. Cells were then washed once and resuspended in PBS. Data were acquired with a BD Biosciences (San Diego, USA) LSR Fortessa X‐20 flow cytometer using an excitation wavelength of 488 nm for DyLight488 and 7AAD and detection wavelengths of 525/50 and 675/20 nm for DyLight488 and 7AAD, respectively. A consistent gating strategy was used as demonstrated in Figure S1. The MFI of mAb labelling was determined using the geometric mean function of FlowJo software v10.7.1 (BD Biosciences). The relative P2X7 expression on cells was determined as the difference between the MFI of anti‐P2X7 mAb and the isotype control mAb. Gating strategies for these and the flow cytometric studies below were based on journal guidelines [37].

### 4.5 Fura‐2 AM Ca^2+^ Response Assay

P2X7 activity was assessed using a Ca^2+^ response assay as described [38]. HEK‐P2X7 and HEK‐293 cells were plated in a black clear‐bottom 96‐well plate (5 × 10^4^ cells per well; Greiner Bio‐One, Krems Master, Austria) overnight at 37°C and 95% air/5% CO_2_. Cells were washed twice using an extracellular Ca^2+^ solution (ECS; 145 mM NaCl, 2 mM CaCl_2_, 1 mM MgCl_2_, 5 mM KCl, 13 mM glucose, and 10 mM HEPES, pH 7.4), then preincubated with Fura‐2 AM loading buffer [2.5‐µM Fura‐2 AM (Thermo Fisher Scientific)/0.2% pluronic acid (Sigma Aldrich, St Louis, USA) in ECS] in the dark for 30 min at 37°C and 95% air/5% CO_2_. Cells were then washed once and incubated with ECS for a further 20 min to allow complete de‐esterification of the Fura‐2 AM. Fura‐2 AM fluorescence was measured at 510 nm every 5 s using a FlexStation 3 (Molecular Devices) following excitation at 340 and 380 nm. Baseline recordings were taken for 30 s, and then following the addition of ATP (Sigma Aldrich) or ECS (basal), recordings were measured for up to 3 min. Where indicated, anti‐P2X7 (clone L4) or isotype control (clone MPC‐11) mAbs were added to wells at regularly spaced 1 min intervals at 37°C for 10 min prior to the addition of ATP or ECS. Data was acquired using SoftMax Pro version 7.0 (Molecular Devices). Relative changes in intracellular Ca^2+^ were calculated using the ratio of Fura‐2 AM fluorescence (F340 nm/380 nm). Ca^2+^ responses were normalised to the baseline recordings using the formula ∆Ca^2+^ = ∆F/Frest = (F − Frest)/Frest, where F is the F340 nm/380 nm ratio in a well at a particular time point and Frest is the mean fluorescence of a well from 0 to 30 s (prior to ATP or ECS addition). Ca^2+^ responses over time were plotted in Prism software v8.0.2 (GraphPad Software, La Jolla, USA). To determine relative P2X7‐mediated Ca^2+^ responses, the area under the curve from 100 to 180 s was calculated with GraphPad Prism. ATP‐induced responses were calculated by subtracting the corresponding basal response. These responses were normalised to the maximal ATP‐induced response within their respective experiment.

### 4.6 CDC Assay (Determined Using LDH Release)

CDC of HEK‐P2X7 and HEK‐293 cells was assessed using a modification of an assay described previously [39]. Cells were plated (5 × 10^4^ cells per well) in a flat‐bottom 96‐well plate (Greiner Bio‐One). Plates were incubated overnight at 37°C and 95% air/5% CO_2_. Cells were washed twice with RPMI‐1640 medium containing 2 mM GlutaMAX and 0.1% bovine serum albumin (BSA; Amresco, Solon, USA; RPMI‐BSA). Then, 50 µL RPMI‐BSA, 50 µL of PBS, anti‐P2X7 or isotype control mAb (2 µg/mL), and 50 µL of rabbit complement (at a final dilution of 1:4) (Cedarlane Laboratories, Burlington, Canada) were added per well in triplicate. To determine maximal LDH release, 135 µL RPMI‐BSA and 15 µL 10x Lysis Solution (Promega, Madison, USA) were added to wells in triplicate (maximum release wells). Plates were incubated for 2 h at 37°C and 95% air/5% CO_2_. Following incubation, 50 µL from all wells were transferred to corresponding wells of a new flat‐bottom 96‐well plate. To three other wells, 50 µL of RPMI‐BSA was added (medium‐only wells). LDH release was assessed using a 96 Non‐Radioactive Cytotoxicity Assay (Promega) according to the manufacturer's instructions. Briefly, a total of 50 µL of CytoTox96 reagent was added per well and plates were incubated for 30 min at room temperature in the dark. Incubations were stopped by the addition of 50 µL of stop solution to each well. Absorbances of each well were read at 490 nm on a SpectraMax Microplate Reader (Molecular Devices) with the mean of medium‐only wells used to define background absorbances, which were subtracted from all other absorbance values. Percent cytotoxicity was determined as the percent of LDH release in sample wells compared with the mean of maximum release wells.

### 4.7 CDC Assay (Using 7AAD Exclusion)

CDC of RPMI 8226 and J774 cells or freshly isolated PBMCs was performed using a variation of the method above. Cells were washed twice (300 × *g* for 5 min) in RPMI‐BSA and resuspended in RPMI‐BSA (3 × 10^6^ cells/mL). Then 150 µL of cell suspension, 150 µL of PBS, anti‐P2X7 or isotype control mAb (2 µg/mL), and 150 µL of rabbit complement (at a final dilution of 1:8) were added per well to a flat‐bottomed 24‐well plate (Greiner Bio‐One). Plates were incubated for 2 h at 37°C 95% air/5% CO_2_. RPMI 8226 and J774 cells were incubated with 7AAD (1 µg/mL) for 5 min at room temperature in the dark. Data were directly acquired from wells with a BD Biosciences Accuri flow cytometer using an excitation wavelength of 488 nm and detection wavelength of 675/20 nm. Percent cytotoxicity was determined using FlowJo v10.7.1 software with the gating strategy demonstrated in Figure S2. PBMCs were scraped and collected from duplicate wells and transferred to 5 mL polystyrene tubes (Sarstedt, Nümbrecht, Germany) and centrifuged (300 × *g* for 5 min). PBMCs were also immunolabelled with fluorochrome‐conjugated mAbs (Table S1), with Precision Count Beads (BioLegend, San Diego, USA) added to each tube. Data were acquired using a BD LSR Fortessa X‐20 flow cytometer at the corresponding excitation and emission wavelengths. Numbers of immune cell subsets were assessed using FlowJo software version 10.7.1. Percent changes in the number of cell subsets were compared between anti‐P2X7 mAb‐treated cells and isotype control mAb‐treated cells.

### 4.8 P2X7 Expression on PBMCs by Immunolabelling and Flow Cytometry

Freshly isolated PBMCs were washed (300 × *g* for 5 min) and resuspended in PBS. Cells (1 × 10^6^ cells/mL) were incubated with Zombie Dye NIR (BioLegend; 1 µg/mL) for 15 min in the dark on ice. Cells were washed with PBS containing 2.5% FCS and centrifuged (300 × *g* for 5 min). Cells were incubated with 10 µL of human FcR Blocking Reagent (Miltenyi Biotec, Bergisch Gladbach, Germany) for 10 min on ice and then with fluorochrome‐conjugated mAbs (Table S1) for 15 min in the dark on ice. Cells were washed twice with PBS. Data were acquired using an LSR Fortessa X‐20 flow cytometer at the corresponding excitation and emission wavelengths (Table S1). The MFI of anti‐P2X7 mAb labelling was determined using the geometric mean function of FlowJo software v10.7.1. The relative P2X7 expression on cells was determined as the difference between the MFI of anti‐P2X7 mAb stained cells and the corresponding FMO control cells.

### 4.9 Data Presentation and Statistical Analysis

Data are represented as the mean ± standard error of the mean (SEM). All statistical analyses were conducted, and graphs were assembled, using GraphPad Prism software v8.0.2. Data were tested for normality using a Shapiro–Wilk test. Statistical differences were calculated using an unpaired (or paired as indicated) Student's *t*‐test (two‐tailed; parametric) or Mann–Whitney test (non‐parametric) for single comparisons or one‐way analysis of variance (ANOVA; parametric) or Kruskal–Wallis (non‐parametric) with a Tukey's post hoc test for multiple comparisons. For all analyses, differences were considered significant if *p *< 0.05.

## Author Contributions

Conceptualisation: Amal Elhage, Debbie Watson, and Ronald Sluyter. Methodology: Amal Elhage, Debbie Watson, and Ronald Sluyter. Formal analysis, investigation, data curation: Amal Elhage. Writing–original draft preparation: Amal Elhage, Debbie Watson, and Ronald Sluyter. Writing–review and editing and visualisation: Amal Elhage. Supervision: Debbie Watson and Ronald Sluyter. Project administration: Ronald Sluyter. Funding acquisition: Debbie Watson and Ronald Sluyter. All authors have read and agreed to the published version of the manuscript.

## Ethics Statement

The study was conducted in accordance with the Declaration of Helsinki, and the protocol approved by the Human Ethics Research Committee of the University of Wollongong (HE12/290, approved 2/11/12) for studies involving humans.

## Consent

Informed consent was obtained from all subjects involved in the study. Written informed consent has been obtained from the donors to publish this paper.

## Conflicts of Interest

The authors declare no conflicts of interest.

## Supporting information



Supplementary information

## Data Availability

The data supporting the findings of this study are available from the corresponding author upon reasonable request.
