# Supplementary material for: The Anti‐Human P2X7 Monoclonal Antibody (Clone L4) Can Mediate Complement‐Dependent Cytotoxicity of Human Leukocytes
Source: Eur J Immunol. 2025 Jan 24;55(1):e202451196. doi: 10.1002/eji.202451196 (PMC11760643; doi:10.1002/eji.202451196)
Supplement: Supplementary file 1 — Supplementary information [file EJI-55-e202451196-s001.pdf]

Supporting information

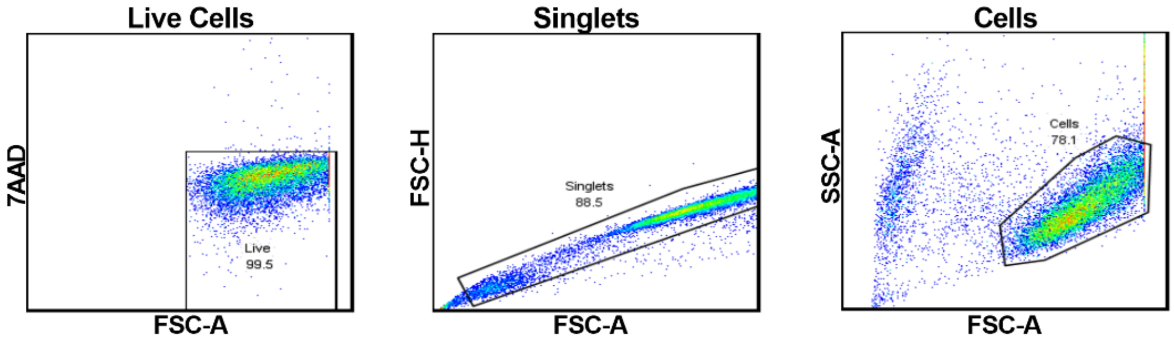

Supplementary Figure 1: Gating strategy used to identify HEK-P2X7 and HEK-293 cells. P2X7 labelled cells were analysed by flow cytometry. Live cells were gated based on forward scatter-area (FSC-A) and 7AAD staining. Singlets were gated based on FSC-A and forward scatter-height (FSC-H). HEK-P2X7 or HEK-293 were gated using FSC-A and side scatter-area (SSC-A).

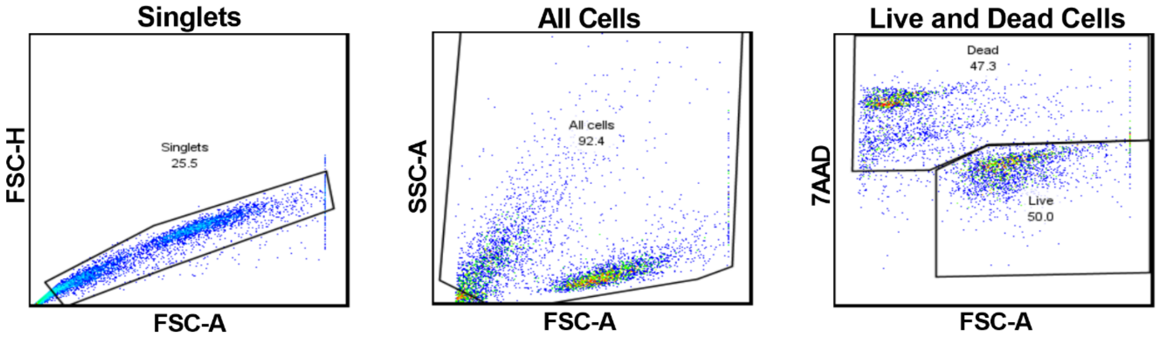

Supplementary Figure 2: Gating strategy used to identify live/dead cells following CDC assays with RPMI 8226 and J774 cells. 7AAD stained cells were analysed by flow cytometry. Singlets were gated based on FSC-A and FSC-H. All cells were gated based on FSC-A and SSC-A. Proportions of live/dead cells were determined by gating on FSC-A and 7AAD.

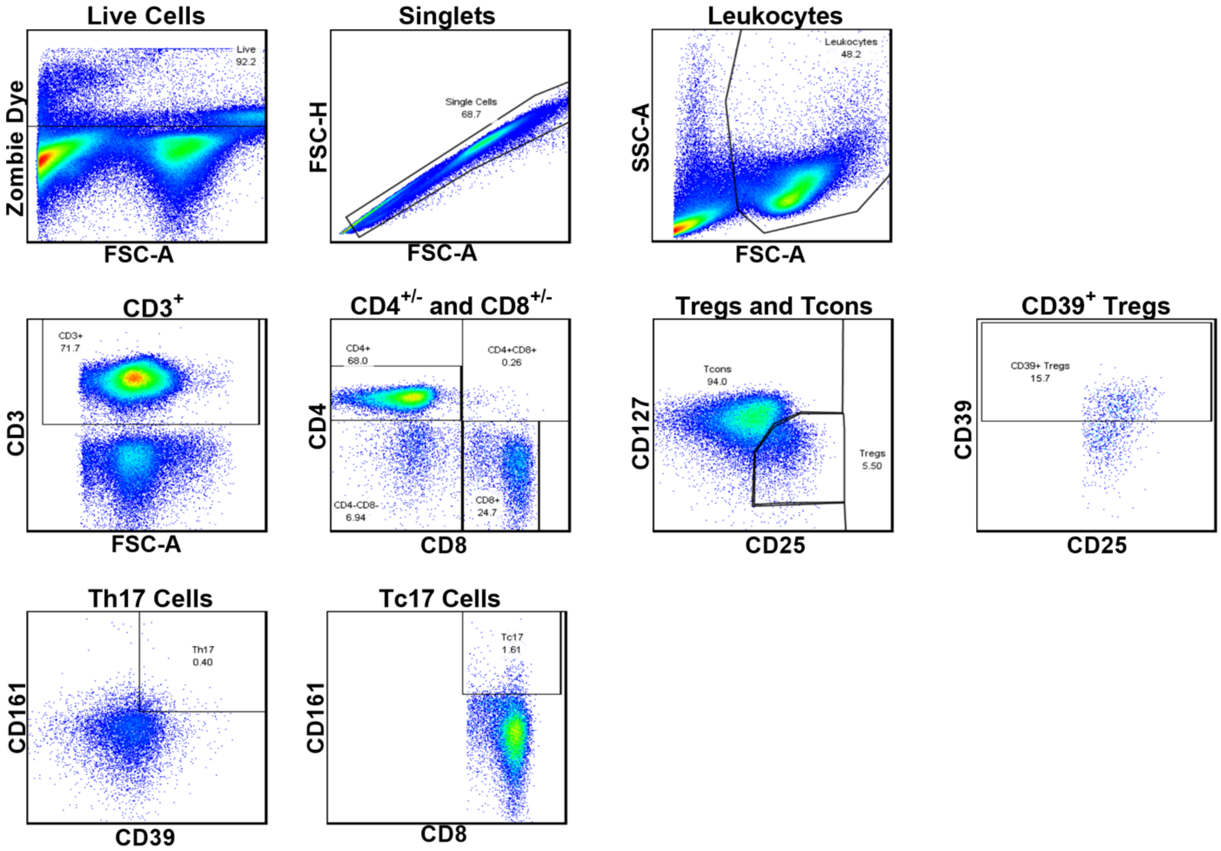

Supplementary Figure 3: Gating strategy used to identify human T cells in P2X7 expression studies and CDC assays. Human leukocyte subsets were analysed by flow cytometry. Live cells were gated based on FSC-A and Zombie NIR staining. Singlets were gated based on FSC-A and FSC-H. The proportions of leukocytes were gated using FSC-A and SSC-A before gating $CD3^+$ T cells, $CD4^{+/-}$ and $CD8^{+/-}$ T cell subsets, $CD4^+CD25^+CD127^{lo}$ Tregs, $CD39^+$ Tregs, $CD4^+CD161^+CD39^+$ Th17 cells and $CD8^+CD161^{hi}$ Tc17 cells.

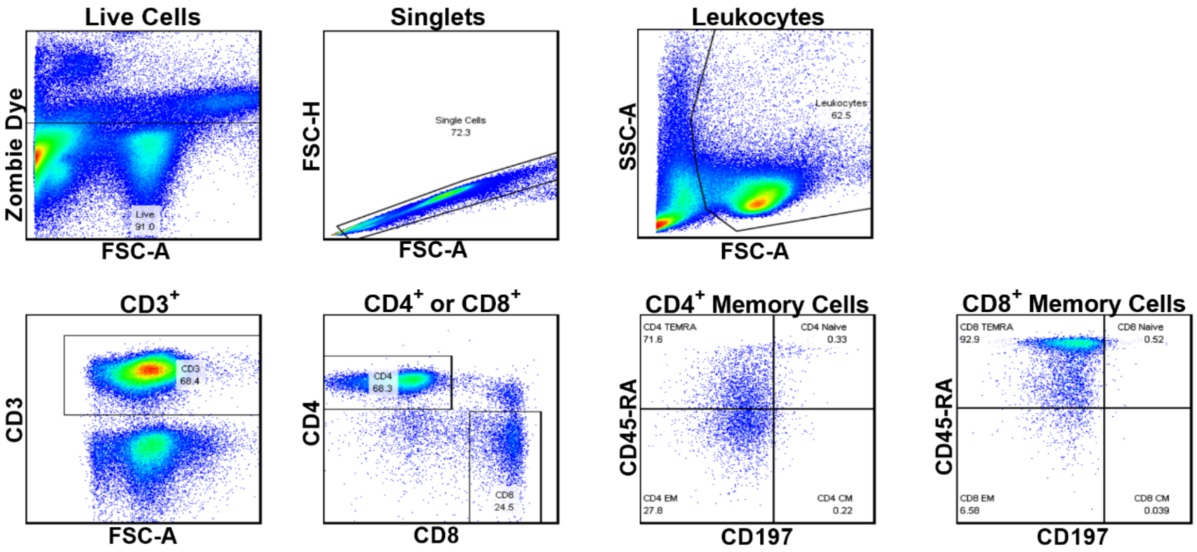

Supplementary Figure 4: Gating strategy used to identify human naive and memory T cells in P2X7 expression studies and CDC assays. Human leukocyte subsets were analysed by flow cytometry. Live cells were gated based on FSC-A and Zombie NIR staining. Singlets were gated based on FSC-A and FSC-H. The proportions of leukocytes were gated using FSC-A and SSC-A before gating CD3$^+$ T cells, CD4$^+$ and CD8$^+$ T cell subsets, CD197$^{+/-}$CD45-RA$^{+/-}$CD4$^+$ naive/memory cells and CD197$^{+/-}$CD45-RA$^{+/-}$CD8$^+$ naive/memory cells.

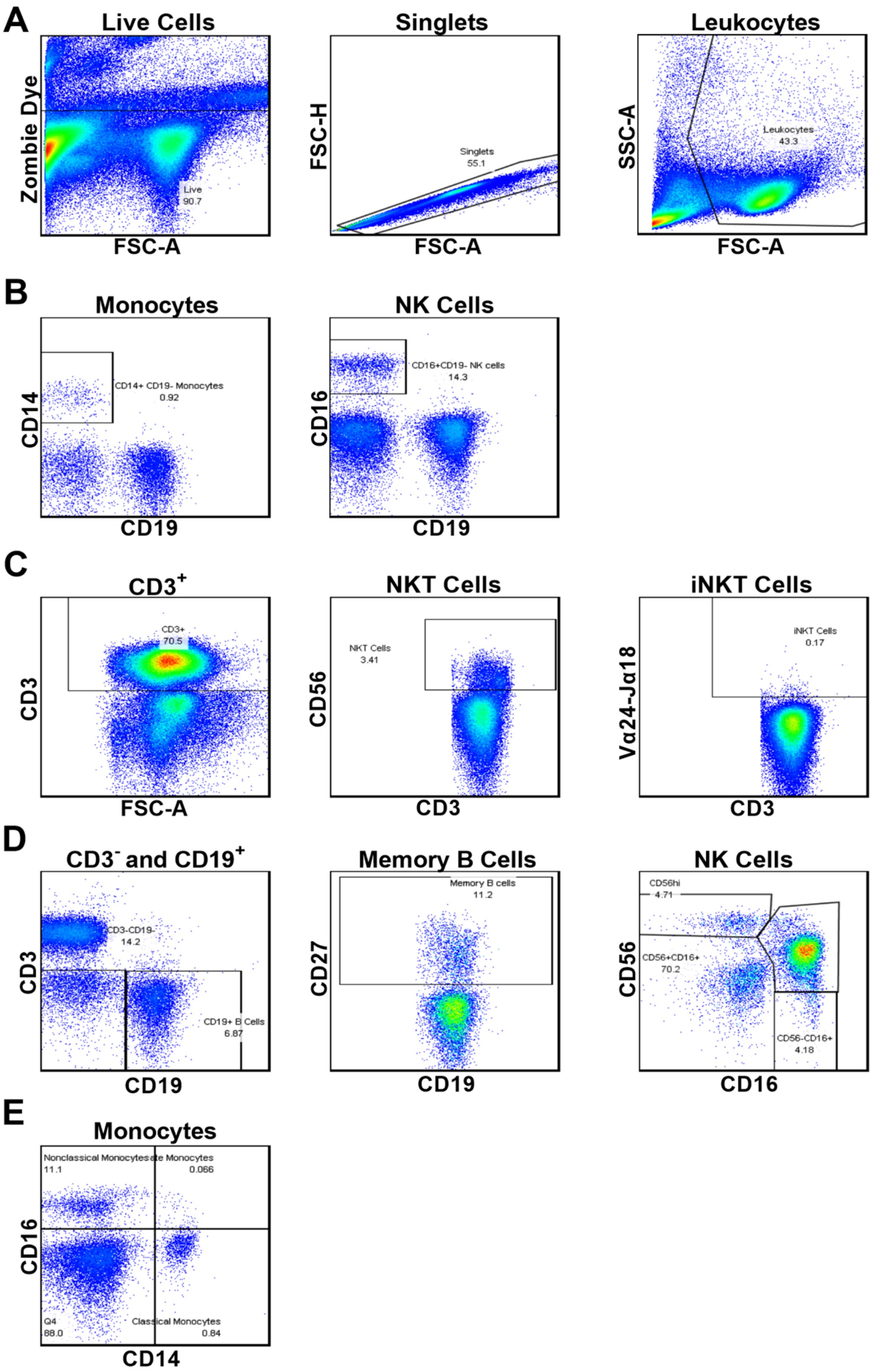
A
Live Cells
Zombie Dye
FSC-A
Live
90.7
Singlets
FSC-H
FSC-A
Singlets
55.1
Leukocytes
SSC-A
FSC-A
Leukocytes
43.3
B
Monocytes
CD14
CD19
CD14+ CD19- Monocytes
0.92
NK Cells
CD16
CD19
CD16+CD19- NK cells
14.3
C
CD3+
CD3
FSC-A
CD3+
70.5
NKT Cells
CD56
CD3
NKT Cells
3.41
iNKT Cells
Vα24-Jα18
CD3
iNKT Cells
0.17
D
CD3- and CD19+
CD3
CD19
CD3-CD19-
14.2
CD19+ B Cells
6.87
Memory B Cells
CD27
CD19
Memory B cells
11.2
NK Cells
CD56
CD16
CD56hi
4.71
CD56+CD16+
70.2
CD56-CD16+
4.18
E
Monocytes
CD16
CD14
Nonclassical Monocytes
11.1
te Monocytes
0.066
Q4
88.0
Classical Monocytes
0.84

Supplementary Figure 5: Gating strategy used to identify human lymphocytes and monocytes in P2X7 expression studies and CDC assays. **(A-D)** Human leukocyte subsets were analysed by flow cytometry. **(A)** Live cells were gated based on FSC-A and Zombie NIR staining. Singlets were gated based on FSC-A and FSC-H. The proportions of leukocytes were gated using FSC-A and SSC-A before gating **(B)** $CD14^{+}CD19^{-}$ monocytes and $CD16^{+}CD19^{-}$ NK cells, **(C)** $CD3^{+}$ T cells, $CD3^{+}CD56^{+}$ NK T cells, $CD3^{+}V\alpha24\text{-}J\alpha18^{+}$ iNKT cells, **(D)** $CD3^{-}CD19^{+}$ B cells, $CD27^{+}$ memory B cells, $CD3^{-}CD19^{-}CD56^{+/-}CD16^{+/-}$ NK cells, **(E)** and $CD16^{+/-}CD14^{+/-}$ monocytes.

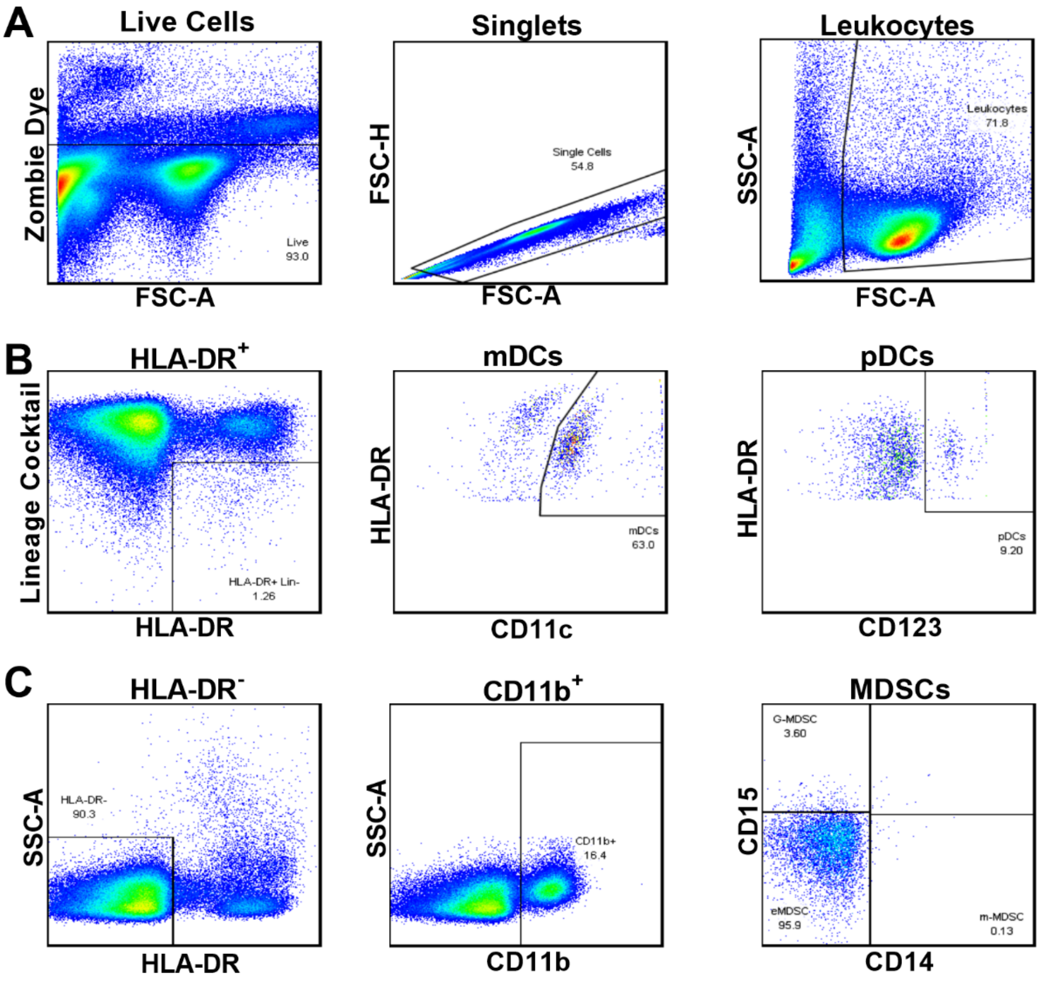

Supplementary Figure 6: Gating strategy used to identify human DCs and MDSCs in P2X7 expression studies and CDC assays. **(A-C)** Human leukocyte subsets were analysed by flow cytometry. **(A)** Live cells were gated based on FSC-A and Zombie NIR staining. Singlets were gated based on FSC-A and FSC-H. The proportions of leukocytes were gated using FSC-A and SSC-A before gating **(B)** lineage cocktail$^-$, HLA-DR$^+$, HLA-DR$^+$CD11c$^+$ mDCs and lineage cocktail$^-$ HLA-DR$^+$CD123$^+$ pDCs. **(C)** or HLA-DR$^-$, CD11b$^+$ and CD14$^{+/-}$CD15$^{+/-}$ MDSCs.

Table S1: Monoclonal antibodies and flow cytometric panels used for human mononuclear leukocyte cells

**Antibody**[a]	**Clone**	**Fluorochrome**[b]	**Dilution**
**Panel 1 – T Cells**			
CD3	UHCT1	BV711	1:50
CD4	RPA-T4	PerCP-Cy5.5	1:10
CD8	RPA-T8	PE-Cy7	1:50
CD25	M-A251	PE	1:20
CD39	TU66	APC	1:20
CD127	HIL-7R-M21	BV421	1:20
CD161	HP-3G10	BV605	1:50
P2X7	L4	DyLight488	1:10
**Panel 2 – Memory T Cells**			
CD3	UHCT1	BV711	1:50
CD4	RPA-T4	PerCP-Cy5.5	1:10
CD8	RPA-T8	PE-Cy7	1:50
CD38	HIT2	APC	1:20
CD45-RA	HI100	PE	1:20
CD197 (CCR7)	150503	BV421	1:20
P2X7	L4	DyLight488	1:10
**Panel 3 – Lymphocytes and Monocytes**			
CD3	UHCT1	BV711	1:50
CD14	MPhiP9	BV421	1:50
CD16	3G8	PerCp-Cy5.5	1:20
CD19	HIB19	APC	1:20
CD27	L128	BV605	1:20
CD56	MY31	PE	1:20
P2X7	L4	DyLight488	1:10
Vα24-Jα18 (iNKT)	6B11	PE-Cy7	1:50
P2X7	L4	DyLight488	1:10
**Panel 4 – DCs and MDSCs**			
CD11b	M1/70	PE-Cy7	1:50

CD11c	B-ly6	BV711	1:50
CD14	MPhiP9	BV421	1:50
CD15	7C3.RMAB	BV605	1:20
CD123	7G3	PE	1:50
HLA-DR	L243	PerCP-Cy5.5	1:20
Lineage cocktail (CD3, 14, 19, 20, 56)	UCHT1; HCD14; HIB19; 2H7; HCD56	APC	1:20
P2X7	L4	DyLight488	1:10

[a] All antibodies, except lineage cocktail and Vα24-Jα18 (BioLegend), were from BD Biosciences.
[b] BV; Brilliant violet, PerCP; peridinin chlorophyll protein-cyanine, PE-Cy; phycoerythrin-cyanine, PE; phycoerythrin and APC; allophycocyanin.
